# BAMT-Net: a boundary-aware multi-task framework for 3D breast ultrasound lesion segmentation and classification

**DOI:** 10.1007/s11517-026-03577-1

**Published:** 2026-04-30

**Authors:** Shuang He, Dan Ji, Mingwei Ma, Xiang Pan, Juxiang Xu, Feng Liu

**Affiliations:** 1 Breast Cervical Center, Jiangyin Hospital of Traditional Chinese Medicine, Wuxi, China; 2 School of Artificial Intelligence and Computer Science, Jiangnan University, Wuxi, China; 3 Department of Medical Imaging, Jiangyin Hospital of Traditional Chinese Medicine, Wuxi, China

**Keywords:** ABVS, Multi-task learning, Lesion segmentation, Breast cancer classification

## Abstract

**Abstract:**

This study introduces an efficient Automated Breast Volume Scanner (ABVS) image analysis framework designed to address the limitations of traditional screening methods-namely low diagnostic accuracy, excessive reliance on operator expertise, and the increased workload associated with manual interpretation of three-dimensional imaging. Our primary objective was to develop a single, end-to-end network that simultaneously performs lesion segmentation and benign-malignant classification using a multi-task learning strategy. The proposed model integrates several innovations. A boundary-aware auxiliary task and a semantic guidance module were designed to enhance the correlation between accurate boundary delineation and classification. We also developed a hybrid weighted attention mechanism that combines spatial and channel-wise information, improving the model’s ability to identify lesions against complex tissue backgrounds. Our findings show that this integrated approach significantly improves performance in both segmentation and classification. By leveraging the intrinsic relationship between the two tasks, the model achieves more precise lesion capture and feature extraction. This framework offers a practical and effective automated solution for clinical breast lesion assessment, enhancing diagnostic accuracy and efficiency while reducing the interpretive burden on radiologists.

**Graphical Abstract:**

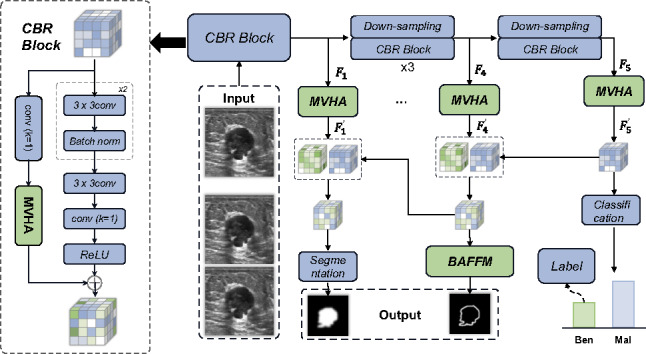

## Introduction

Mammography and ultrasound have long been the primary modalities for breast cancer screening; however, both methods still have certain limitations. In mammography, high breast density can sometimes obscure the visualization of lesions [1, 2]. While ultrasound is effective for displaying certain types of lesions, its heavy reliance on the operator’s experience can lead to diagnostic discrepancies. Unlike the standardized position and fixed parameters of mammography, handheld ultrasound scanning coverage, probe pressure, and gain adjustment vary from person to person, making complete standardization difficult. The image quality and diagnostic information acquisition are highly dependent on the operator’s scanning technique, instrument adjustment ability, and real-time interpretation skills [3, 4].Furthermore, the two-dimensional images from traditional imaging techniques are often constrained by resolution and viewing angles, particularly when visualizing small or deep-seated lesions. Automated Breast Volume Scanner (ABVS), a novel three-dimensional breast imaging technology, is emerging as an increasingly popular choice [5]. Compared to traditional ultrasound, ABVS employs advanced 3D imaging methods to provide a comprehensive view of the internal breast structure, clearly presenting details of lesions, organ boundaries, and other abnormal areas [6, 7]. During examinations, ABVS utilizes a wide-range high-frequency probe controlled by a robotic arm to automatically scan the breast along a preset path, eliminating the need for manual probe handling and movement by the operator [8, 9]. This technology significantly reduces operator dependency and effectively prevents missed diagnoses caused by inexperience. This helps clinicians make more accurate diagnoses with more comprehensive and intuitive data, significantly improving screening accuracy and the reliability of early diagnosis [10–13].

However, while the 3D images acquired by ABVS provide richer and more detailed information about lesions compared to 2D images, they also geometrically increase the workload for radiologists. Firstly, 3D images consist of hundreds to thousands of slices, resulting in a data volume far exceeding that of 2D images. This directly leads to a significant increase in workload, requiring longer time for image processing, 3D reconstruction, and detailed interpretation [14, 15]. Secondly, interpreting 3D images in virtual space requires radiologists to possess higher depth perception and spatial cognitive abilities to accurately understand the spatial relationship of anatomical structures and lesions. Prolonged handling of complex 3D data, particularly in the absence of high-quality visualization tools, can easily lead to visual fatigue and decreased attention, thus compromising diagnostic efficiency and accuracy [16].

Fortunately, the rapid development of artificial intelligence (AI) offers a new solution to this challenge. By constructing an intelligent diagnostic model for preliminary screening and automatic delineation of suspicious lesions in ABVS data, an efficient screening workflow can be established, with clinicians performing the final verification. Compared to the traditional “double reading” method, this AI-assisted approach not only significantly enhances the efficiency of early screening and alleviates the workload of clinicians but also effectively reduces fatigue from prolonged image review, thereby minimizing the risk of misdiagnosis and missed detections.

With the rapid advancement of AI, a growing number of models for processing 3D medical images have emerged. The most advanced methods for 3D ABUS analysis predominantly adopt sophisticated architectures, including hybrid models (e.g., 3D Swin-UNETR variants) that combine CNNs and Transformers to capture both local detail and global context, or utilize robust frameworks like 3D nn-UNet as performance baselines. Furthermore, the latest trends involve exploring generalist medical Foundation Models (e.g., MedSAM2) for enhanced generalization. Most of these models follow a two-stage process of segmentation followed by classification, where lesions are first segmented and then diagnosed to obtain the final result. However, this two-stage approach requires the construction of two separate networks, which is not only inefficient but also computationally demanding, making it difficult to implement in practical clinical scenarios.

To address this issue, we have developed an integrated framework for segmentation and classification of ABVS images based on a multi-task learning strategy. The objective is to simultaneously achieve both lesion segmentation and benign-malignant classification in 3D medical images within a single end-to-end network. This approach aims to fully leverage the intrinsic correlations between the tasks, allowing them to mutually reinforce each other and enhance overall performance.

## Method

### Ethics approval

This study was approved by the Ethics Committee of Jiangyin Traditional Chinese Medicine Hospital. The requirement for written informed consent was waived by the committee due to the retrospective nature of the study.

### Dataset and data processing

In this study, we utilize two distinct datasets for training and evaluation: a public challenge dataset and a private clinical dataset. This dual-dataset approach allows us to validate the robustness and generalization capability of our proposed model. To address the confusion regarding the total case count, we provide a detailed explanation of the data combination and processing steps below.

#### Data sources and preparation

We first utilize the TDSC-ABUS [5] (Tumor Detection, Segmentation and Classification Challenge on Automated 3D Breast Ultrasound) dataset. This dataset, originating from the Harbin Medical University Cancer Hospital, contains 200 high-resolution ABUS scans, comprising 110 benign and 90 malignant cases. The image dimensions in this dataset range from $843 \times 546 \times 270$ to $865 \times 682 \times 354$ pixels, with a pixel spacing of $0.2 \text {mm} \times 0.073 \text {mm}$ and a slice spacing of $0.47 \text {mm}$.

In addition to the public dataset, we collected a retrospective clinical cohort from the Jiangyin Hospital of Traditional Chinese Medicine, comprising 989 screening cases from January 2023 to December 2023. These cases were initially assessed and categorized according to the Breast Imaging Reporting and Data System (BI-RADS) guidelines. From this initial cohort, we implemented a strict screening process to ensure data quality and relevance. Specifically, we first excluded all cases with a BI-RADS classification less than 4 (i.e., BI-RADS 1, 2, and 3) to focus on suspicious lesions. We then further selected only those cases that had received pathological examination (biopsy or surgery) at our affiliated institution to confirm the benign or malignant status. The corresponding ultrasound images for the selected cases were subsequently annotated by two experienced radiologists. This rigorous selection process resulted in a final cohort of 400 valid cases from the private data, including 145 benign and 255 malignant lesions.

#### Combined dataset partition

The final experimental dataset combines the 200 cases from TDSC-ABUS and the 400 selected cases from the private clinical cohort, resulting in a total of 600 cases. Both TDSC-ABUS and the private cohort cases were subjected to malignancy confirmation to ensure consistency. The combined 600 cases were then subjected to a strict stratified sampling strategy to create the Training and Test sets, ensuring that the proportions of benign and malignant cases were maintained across the partitions.

The overall distribution of the two data sources and the partitioning of the total dataset are detailed in Table 1.

**Table 1:** Summary of data sources, malignancy status, and final experimental partition (Total 600 Cases)

Partition / Source	TDSC-ABUS (200)		Private Cohort (400)		Total Cases
	Benign	Malignant	Benign	Malignant	
Training Set	82	68	95	202	447
Test Set	28	22	50	53	153
Source Total	**110**	**90**	**145**	**255**	**600**

### A multi-task architecture for analyzing automated breast volume scanner images

**Fig. 1:**
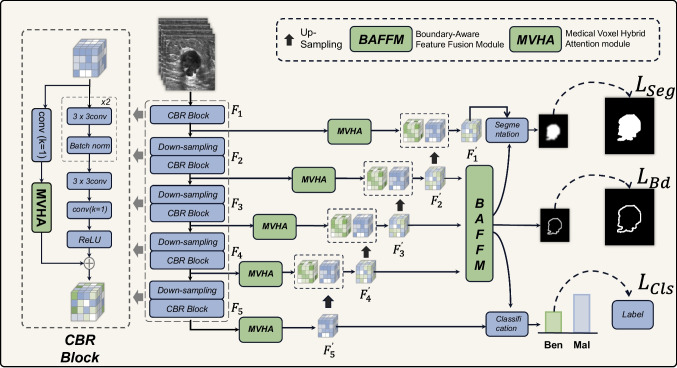
Schematic diagram of multitasking architecture for analyzing full-volume mammary ultrasound images

In this study, we propose a novel boundary semantic information-guided multi-task learning architecture (as shown in Fig. 1) specifically designed for the analysis of ABVS images. This architecture employs a unified 3D convolutional network model to simultaneously perform two critical tasks: tumor region segmentation and benign-malignant classification. It fully leverages the complementary information between these tasks to significantly enhance the model’s performance on each. Furthermore, it utilizes the morphological boundary and contour information of the tumor to guide the model, deepening its understanding of the lesion area to further improve both segmentation and classification performance.

In summary, the entire multi-task architecture consists of three core components: an encoder, a Boundary-Aware Feature Fusion Module (BAFFM), and a Medical Voxel Hybrid Attention (MVHA) module. In the following sections, we will provide a detailed introduction to each of these three parts.

### Encoder network architecture

**Fig. 2:**
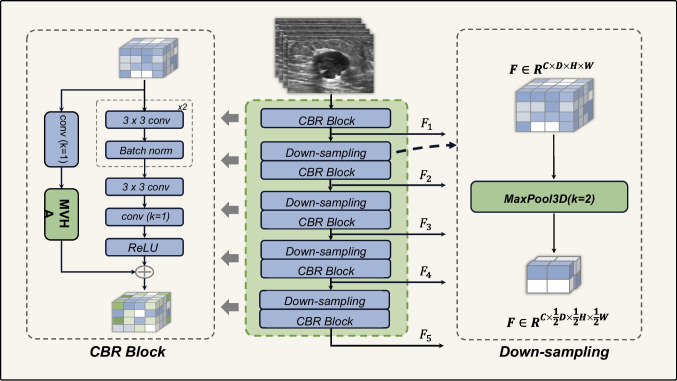
Encoder network structure

The encoder network architecture is illustrated in Fig. 2. For the encoder design, we adopted a feature extraction network composed of five CBR (Convolution-Batch Normalization-ReLU) modules and four down-sampling modules to thoroughly mine the deep-level information from breast cancer ultrasound images. Within the CBR module, feature extraction is split into two branches to enhance the ability to extract diverse features. The specific implementation is as follows: In the first branch, the input feature *F* passes through the CBR module, where it first undergoes two feature extraction layers, each consisting of a $3\times 3\times 3$ 3D convolution kernel and batch normalization, to progressively learn local spatial features. Subsequently, this feature passes sequentially through a $3\times 3\times 3$ convolutional layer, a $1\times 1\times 1$ convolutional layer, and a ReLU activation function to obtain more refined conventional features, denoted as $F_1$. In the second branch, the input feature *F* first passes through a $1\times 1\times 1$ convolutional layer for feature extraction to capture more compact spatial information. It is then further processed by the MVHA attention module to enhance the network’s focus on features relevant to the lesion area, thereby generating feature $F_2$. Finally, the features $F_1$ and $F_2$ from these two branches are fused via element-wise addition, resulting in a deeper feature representation $F'$ compared to the original input feature *F*. After this process, the feature $F'$ undergoes a down-sampling operation to further reduce its dimensionality before being passed to the next CBR module, thus gradually building a richer feature representation. Through this meticulously designed, simple yet efficient encoder structure, we not only optimize local spatial information extraction but also effectively enhance the model’s perceptual ability for lesion areas by integrating the attention mechanism.

During the down-sampling process, we employ a max-pooling function to reduce the dimensionality of the features. This effectively minimizes redundancy while preserving the critical features essential for the tasks. Our model uses a down-sampling rate of 2 (i.e., max-pooling with a stride of 2) to progressively reduce the spatial dimensions of the feature maps while maintaining important structural information. Specifically, after each down-sampling operation, the depth, height, and width of the feature map are halved, while the number of channels remains unchanged. This process not only reduces the computational complexity, allowing subsequent network layers to process information more efficiently, but also highlights significant regions in the breast cancer images, enhancing the model’s ability to perceive key structures of lesion areas. Through max-pooling, the network preserves as much detail of the target area as possible while reducing data dimensionality, laying a solid foundation for subsequent feature extraction and task optimization.

### Medical Voxel Hybrid Attention (MVHA) Module

In traditional computer vision tasks, spatial and channel attention mechanisms have demonstrated remarkable effectiveness by enhancing the network’s focus on target regions, thereby optimizing feature extraction and object recognition performance. However, unlike the 2D images processed in these tasks, ABVS images are 3D medical images with a more complex data structure, containing richer spatial information and voxel-level features. Consequently, directly applying existing vision models to medical image analysis often fails to achieve ideal results, primarily because traditional attention mechanisms cannot fully leverage the global feature relationships in 3D images, leading to limited accuracy in lesion recognition and segmentation.

**Fig. 3:**
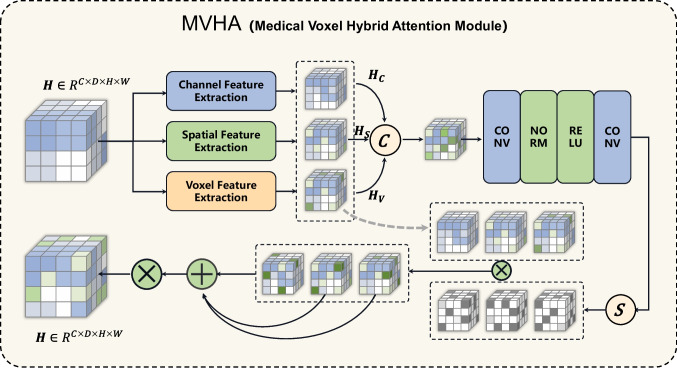
Medical Voxel Hybrid Attention (MVHA) Module

To address this challenge, we propose a hybrid weighted attention mechanism that fuses channel, spatial, and voxel features to fully exploit the multi-dimensional information in ABVS images, thereby improving the network’s performance in both segmentation and classification tasks. By introducing this innovative attention mechanism, the model can more accurately capture the key features of breast lesion areas and enhance its ability to identify diseased tissue, thus playing a more significant role in breast cancer detection and diagnosis. The structure of the MVHA module is shown in Fig. 3.

For a given 3D input feature $H \in \mathbb {R}^{C \times D \times H \times W}$, it is processed through three parallel branches: a channel feature extraction branch, a spatial feature extraction branch, and a voxel feature extraction branch. The processes of these three branches are introduced sequentially below.

#### Channel feature extraction branch

**Fig. 4:**
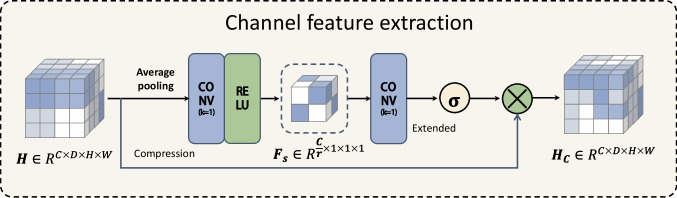
Channel Feature Extraction Branch

In this process, the input feature *H* first undergoes global average pooling for feature dimensionality reduction. This operation effectively extracts global information while retaining only the channel dimension, thereby aggregating channel-level information across the entire feature map (Fig. 4). Subsequently, the feature passes through a channel-reduction convolutional layer and a ReLU activation function. The main purpose of this stage is to perform feature compression along the channel dimension, which highlights key information and effectively removes redundant features, enabling the model to focus on more discriminative channel features. This process can be formulated as follows:

First, global average pooling is applied to the input feature to retain only the channel dimension:

1 $$\begin{aligned} F_c(c) = \frac{1}{D \times H \times W} \sum _{k=1}^{D} \sum _{j=1}^{H} \sum _{i=1}^{W} H(c, k, j, i) \end{aligned}$$

where $F_c \in \mathbb {R}^{C}$ represents the channel-level global feature vector. Then, channel information is compressed through a channel-reduction convolutional layer and a ReLU activation function:

2 $$\begin{aligned} F_s = \text {ReLU}(W_1 F_c + b_1) \end{aligned}$$

where $W_1 \in \mathbb {R}^{(C/r) \times C}$ is the weight of the channel-reduction convolution, $b_1$ is the bias term, and *r* is the channel reduction ratio.

Subsequently, the compressed feature is processed by a channel-expansion convolutional layer, enabling an information “leap” in the channel dimension. This allows the model to recover and reconstruct key features in a higher dimension, enhancing the richness and diversity of the feature representation. This process not only improves the model’s ability to capture important channel features but also enhances its representation capability for ABVS images. Finally, the processed feature is normalized by a Sigmoid function to generate the channel attention weight matrix *W*, formulated as:

3 $$\begin{aligned} W = \sigma (W_2 F_s + b_2) \end{aligned}$$

where $W_2 \in \mathbb {R}^{C \times (C/r)}$ is the weight of the channel-expansion convolution, $b_2$ is the bias term, and $\sigma (\cdot )$ denotes the Sigmoid function, ensuring that the weight values in the channel attention matrix are within the range (0, 1).

The channel attention weight matrix *W* is then multiplied channel-wise with the original input feature *H* to obtain the final channel feature $H_C$. This enables the network to focus more accurately on the channel information that is crucial for the classification and segmentation tasks. The process is formulated as:

4 $$\begin{aligned} H_C = W \odot H \end{aligned}$$

where $\odot$ denotes element-wise channel weighting.

#### Spatial feature extraction branch

**Fig. 5:**
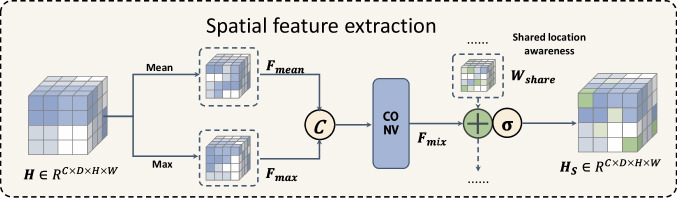
Spatial Feature Extraction Branch

In this process, max-pooling and average-pooling operations are first performed on the input feature *H* along the channel dimension, yielding two feature representations, denoted as $F_{\text {max}}$ and $F_{\text {mean}}$, respectively (Fig. 5). These two features preserve information at different levels: max-pooling highlights the most significant feature regions in the image, while average-pooling captures overall global information, resulting in a more balanced feature representation. This process is formulated as follows:

5 $$\begin{aligned}&F_{\text {max}} = \max _{\text {c}}(H) \end{aligned}$$

6 $$\begin{aligned}&F_{\text {mean}} = \frac{1}{C} \sum _{c=1}^{C} H(c, k, j, i) \end{aligned}$$

where $$F_{\text {max}} \in \mathbb {R}^{1 \times D \times H \times W}$$ and $$F_{\text {mean}} \in \mathbb {R}^{1 \times D \times H \times W}$$.

Next, $F_{\text {max}}$ and $F_{\text {mean}}$ are concatenated along the channel dimension to fuse the two different feature representations. This combined feature is then processed by a large-kernel convolutional layer ($7\times 7\times 7$) to obtain a fused feature representation, $F_{\text {mix}}$. This step expands the model’s receptive field, enabling it to better capture spatial relationships and structural information within the image. The process is formulated as:

7 $$\begin{aligned} F_{\text {mix}} = \text {Conv}_7(\text {Concat}(F_{\text {max}}, F_{\text {mean}}, \text {dim}=C)) \end{aligned}$$

where $\text {Conv}_7(\cdot )$ represents a $7\times 7\times 7$ convolution operation, and $\text {Concat}(\cdot )$ denotes concatenation along the channel dimension.

Additionally, we introduce a globally shared, position-aware weight matrix $W_{\text {share}}$, which is adapted to the spatial dimensions of the current input feature via an interpolation operation to ensure that positional information is fully aligned with the features. This position-aware matrix is then added element-wise to $F_{\text {mix}}$, enhancing the spatial feature with both global and local information from the original input and strengthening its perception of spatial structures. Finally, after normalization by a Sigmoid function, the final spatial attention feature $H_S$ is generated. This process is formulated as follows:

8 $$\begin{aligned} H_S = \sigma (F_{\text {mix}} + \text {Interpolate}(W_{\text {share}}, \text {size}=(D, H, W))) \end{aligned}$$

where $\text {Interpolate}(\cdot )$ denotes the interpolation operation that matches the spatial dimensions of $W_{\text {share}}$ to the current feature, and $\sigma (\cdot )$ denotes the Sigmoid function.

#### Voxel feature extraction branch

**Fig. 6:**
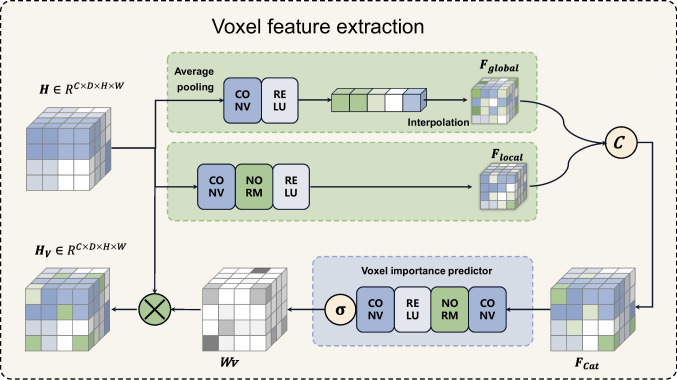
Voxel Feature Extraction Branch

In this process, both global and local information are extracted from the input feature *H* to enhance the model’s perception of features at different scales (Fig. 6).

To extract global information, average pooling is first applied to *H* along the spatial dimensions, retaining only the global feature representation in the channel dimension. Then, a channel-reduction convolutional layer combined with a ReLU activation function is used to further refine the global features, removing redundant information while enhancing the network’s perception of important features. Subsequently, to ensure the global features align with the spatial scale of the original input, an interpolation operation is used to restore the feature to its original dimensions, yielding the global feature representation $F_{\text {global}}$. This is formulated as:

9 $$\begin{aligned}&F_{\text {avg}} = \frac{1}{D \times H \times W} \sum _{k=1}^{D} \sum _{j=1}^{H} \sum _{i=1}^{W} H(:, k, j, i) \end{aligned}$$

10 $$\begin{aligned}&F_{\text {global\_reduced}} = \text {ReLU}(\text {Conv}_{c/r}(F_{\text {avg}})) \end{aligned}$$

11 $$\begin{aligned}&F_{\text {global}} = \text {Interpolate}(F_{\text {global\_reduced}}, \text {size}=(D, H, W)) \end{aligned}$$

where $F_{\text {avg}} \in \mathbb {R}^{C \times 1 \times 1 \times 1}$, $F_{\text {global\_reduced}} \in \mathbb {R}^{C/r \times 1 \times 1 \times 1}$, $\text {Conv}_{c/r}$ represents a convolutional layer that reduces the channel count by a factor of *r*, and $F_{\text {global}} \in \mathbb {R}^{C \times D \times H \times W}$.

In contrast, the extraction of local features focuses more on the detailed information within the image. The input feature *H* is directly processed through a stack of layers consisting of a convolutional layer, a batch normalization layer, and a ReLU activation function to obtain the local feature representation $F_{\text {local}}$. This structure leverages the local receptive field of the convolutional layer to capture fine-grained information, while batch normalization improves the model’s stability and training efficiency. This is formulated as:

12 $$\begin{aligned} F_{\text {local}} = \text {ReLU}(\text {BN}(\text {Conv}(H))) \end{aligned}$$

After extracting the global feature $F_{\text {global}}$ and the local feature $F_{\text {local}}$, they are concatenated along the channel dimension to create a fused feature, $F_{\text {cat}}$, which contains a richer feature representation. This fused feature is then processed by a Voxel Importance Predictor to adaptively learn the weight distribution of each voxel in the feature representation. The Voxel Importance Predictor consists of a series of network layers, including convolutions, batch normalization, and ReLU activations, enabling the network to retain key voxel information based on its importance. Specifically, after passing through the predictor, $F_{\text {cat}}$ generates a voxel-level weight matrix $W_v$, which assigns different importance values to different voxels. This allows the model to focus more precisely on critical regions, further enhancing its ability to analyze ABVS images.

### Boundary-Aware Feature Fusion Module (BAFFM)

**Fig. 7:**
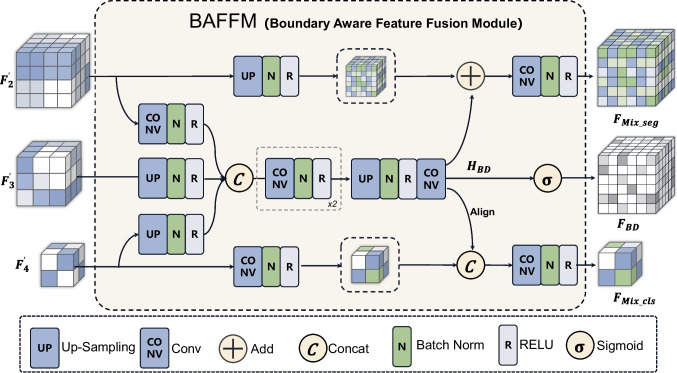
Boundary-Aware Feature Fusion Module (BAFFM)

During the up-sampling process, the resulting features $F_2'$, $F_3'$, and $F_4'$ contain semantic information at different levels. Among them, $F_4'$ is the feature closest to the classification branch and its representation is more akin to class information, enabling it to capture global semantic features effectively. In contrast, $F_2'$, being the feature closest to the segmentation branch, retains richer spatial and structural information, which is particularly crucial for delineating the edges and details of the target. To fully integrate these two types of information, we have designed a feature fusion module to extract more discriminative boundary features, thereby synergistically optimizing the model’s overall performance on both classification and segmentation tasks. Through a multi-level information interaction mechanism, this module combines high-level semantic information with low-level spatial information, allowing the model to maintain its class-discriminative ability while more accurately capturing the boundary features of the target (Fig. 7). Taking an input data size of (128, 128, 128) as an example, the features obtained during the up-sampling process are: $F_4' \in \mathbb {R}^{64 \times 16 \times 16 \times 16}$, $F_3' \in \mathbb {R}^{32 \times 32 \times 32 \times 32}$, and $F_2' \in \mathbb {R}^{16 \times 64 \times 64 \times 64}$. When these features are input into the module, they are processed through three main branches: a Segmentation Auxiliary Branch (top branch in Fig. 5), a Boundary Fusion Branch (middle branch in Fig. 5), and a Classification Auxiliary Branch (bottom branch in Fig. 5).

#### Boundary fusion branch

The input features ($F_4'$, $F_3'$, and $F_2'$) are first spatially aligned to a uniform size of (64, 64, 64) to ensure seamless fusion. The aligned features are then concatenated along the channel dimension and processed by two CBR modules for deep feature extraction. The extracted features are subsequently up-sampled via transposed convolution, followed by a series of operations including a batch normalization layer and a final convolution, to obtain the boundary information feature, $H_{BD}$. Finally, this feature passes through a Sigmoid function to output the predicted tumor boundary, $F_{BD}$, which is subsequently used to calculate the boundary segmentation loss, $L_{Bd}$.

#### Segmentation auxiliary branch

**Fig. 8:**
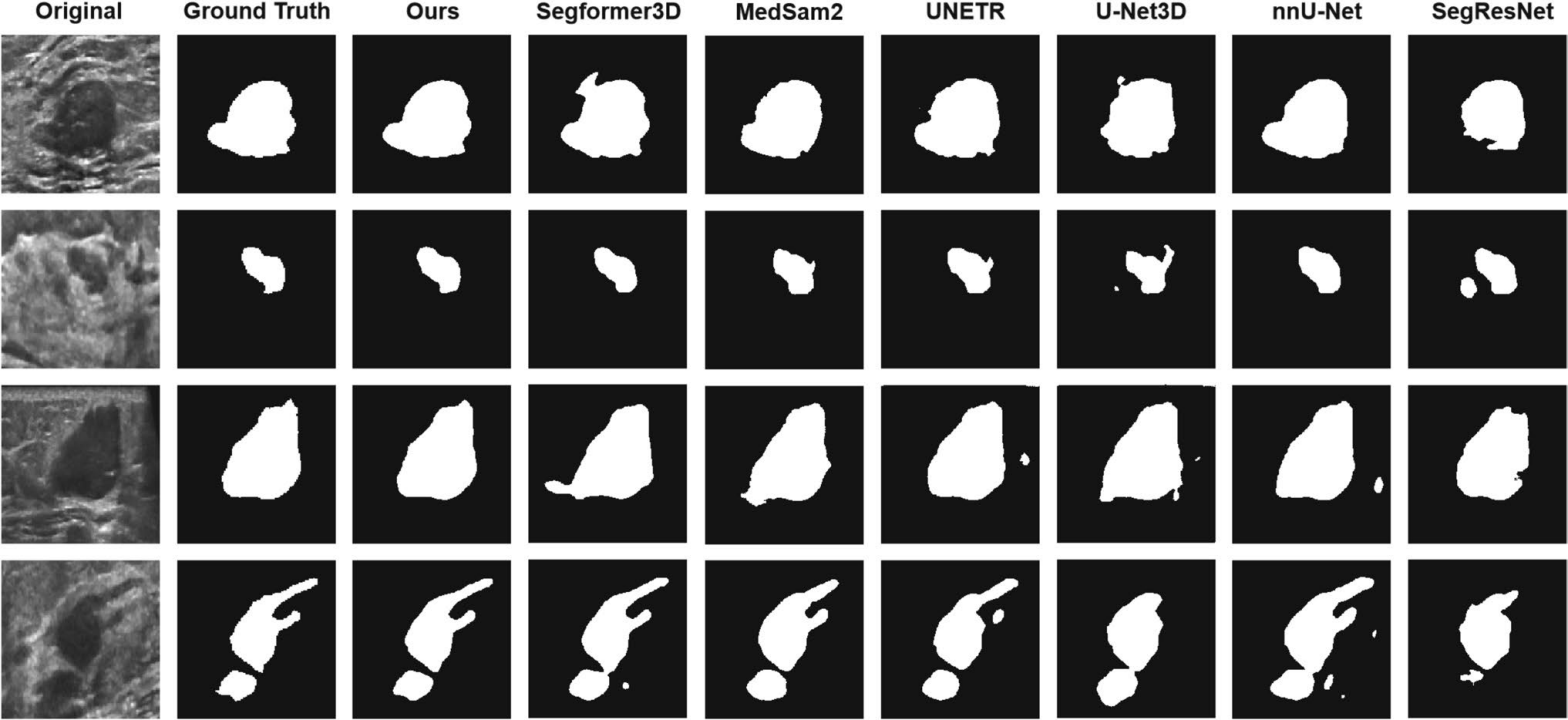
Model segmentation effect comparison

The feature first passes through an up-sampling module, followed by a batch normalization layer and a ReLU activation function to further enhance the expressive power of the segmentation features. Next, the feature from this branch is added element-wise with the boundary feature $H_{BD}$, leveraging boundary information to optimize segmentation performance. Finally, the fused feature is passed through a CBR module for feature alignment, yielding the segmentation auxiliary feature $F_{\text {Mix\_seg}}$. This auxiliary feature is then fed into the segmentation head along with other features to predict the final tumor segmentation result (Fig. 8).

#### Classification auxiliary branch

The input feature undergoes initial feature extraction via a CBR module. The resulting feature is then concatenated along the channel dimension with a down-sampled version of the boundary feature $H_{BD}$. This step aims to fuse classification information with boundary information. The concatenated feature is then passed through another CBR module to align and fuse the classification and boundary information in the semantic space, ultimately generating a classification auxiliary feature, $F_{\text {Mix\_cls}}$. This feature is subsequently fed into the classification head along with other features to perform breast tumor classification. Through this branched feature fusion approach, the model can effectively integrate segmentation, boundary, and classification information, enabling cross-talk and mutual reinforcement between different tasks. This ultimately leads to an overall improvement in the model’s performance on both classification and segmentation of medical images.

### Loss function design

The proposed BAMT-Net operates as a multi-task learning framework designed to simultaneously perform three distinct tasks: lesion segmentation, boundary prediction, and benign/malignant classification. Accordingly, the model’s total loss function, $L_{\text {Total}}$, is defined as a weighted summation of the individual task losses:

13 $$\begin{aligned} L_{\text {Total}} = \alpha L_{\text {Seg}} + \beta L_{\text {Bd}} + \gamma L_{\text {Cls}} \end{aligned}$$

where $L_{\text {Seg}}$ is the segmentation loss, $L_{\text {Bd}}$ is the boundary-aware loss, and $L_{\text {Cls}}$ is the classification loss. $\alpha$, $\beta$, and $\gamma$ are hyperparameters used to balance the contribution of the three tasks. Considering the importance of these tasks, the weighting factors $\alpha$, $\beta$, and $\gamma$ are empirically set to 0.4, 0.2, and 0.4, respectively.

#### Segmentation loss $L_{\text {Seg}}$ and boundary-aware loss $L_{\text {Bd}}$

To effectively address the severe imbalance between positive and negative samples common in breast ultrasound image analysis, both the segmentation loss $L_{\text {Seg}}$ and the boundary-aware loss $L_{\text {Bd}}$ utilize the Dice Loss ($L_{\text {Dice}}$). $L_{\text {Seg}}$ measures the similarity between the predicted segmentation mask $\hat{Y}_{\text {Seg}}$ and the ground truth $Y_{\text {Seg}}$. $L_{\text {Bd}}$ guides the model to learn the boundary morphological features by measuring the similarity between the predicted boundary $\hat{Y}_{\text {Bd}}$ (output of the BAFFM boundary branch) and the ground truth boundary $Y_{\text {Bd}}$.

The precise definition of the Dice Loss ($L_{\text {Dice}}$) is given by:

14 $$\begin{aligned} L_{\text {Dice}}(P, G) = 1 - \frac{2 \sum _{i} (P_i \cdot G_i) + \epsilon }{\sum _{i} P_i + \sum _{i} G_i + \epsilon } \end{aligned}$$

where *P* is the predicted result ($\hat{Y}_{\text {Seg}}$ or $\hat{Y}_{\text {Bd}}$), *G* is the ground truth label ($Y_{\text {Seg}}$ or $Y_{\text {Bd}}$), *i* iterates over all voxels, and $\epsilon$ is a small smoothing term (e.g., $10^{-5}$) to ensure gradient stability.

##### Boundary label $Y_{\text {Bd}}$ generation

The ground truth boundary label $Y_{\text {Bd}}$ is generated from the binary segmentation mask $Y_{\text {Seg}}$ via a multi-step morphological pipeline implemented slice-by-slice. To ensure a high-quality supervision signal, we first apply a morphological opening operation (using a $3 \times 3$ kernel) to eliminate small noise artifacts and smooth the mask edges. Subsequently, the findContours algorithm is utilized to extract the precise coordinates of the lesion perimeter. To further refine the boundary and ensure it accurately represents the transition zone between the tumor and surrounding tissue, a sequence of dilation and erosion operations is applied to the extracted contours. These 2D boundary maps are then concatenated into a 3D volume. This approach means the boundary supervision is unsupervised with respect to external dedicated expert annotations, as it is solely derived from the existing segmentation labels. The process can be summarized as:

15 $$\begin{aligned} Y_{\text {Bd}} = \text {Refine}(\text {Contours}(\text {Open}(Y_{\text {Seg}}))) \end{aligned}$$

#### Classification loss $L_{\text {Cls}}$

For the benign vs. malignant binary classification task, the classification loss $L_{\text {Cls}}$ uses the standard Binary Cross-Entropy (BCE) Loss:

16 $$\begin{aligned} L_{\text {Cls}} = - \left[ Y_{\text {Cls}} \log (\hat{Y}_{\text {Cls}}) + (1 - Y_{\text {Cls}}) \log (1 - \hat{Y}_{\text {Cls}}) \right] \end{aligned}$$

where $Y_{\text {Cls}}$ represents the ground truth label (1 for malignant, 0 for benign), and $\hat{Y}_{\text {Cls}}$ is the predicted probability for the malignant class.

## Results

### Data preprocessing and augmentation

To mitigate the risk of overfitting and enhance model generalization, a consistent preprocessing and augmentation strategy was applied to both datasets. First, a 3-fold data augmentation was performed. The specific strategy was as follows: while ensuring the lesion area remained intact, all data were randomly cropped and then uniformly resized to a standard dimension of (128, 128, 128) to maintain consistency in input data size. The augmented data were then divided into training and test sets following a strict partitioning principle: augmented samples originating from the same raw data were allocated entirely to either the training set or the test set. This approach prevents data leakage and ensures a fair and reliable model evaluation process.

### Performance evaluation metrics

We used the Dice Score, Intersection over Union (IoU), and 95% Hausdorff Distance (HD95) to evaluate segmentation performance. The Dice Score measures the similarity between the predicted segmentation region and the ground truth region. Its value ranges from [0, 1], with values closer to 1 indicating better model performance. The formula is as follows:

17 $$\begin{aligned} \text {Dice} = \frac{2 \times |A \cap B|}{|A| + |B|} \end{aligned}$$

where *A* represents the ground truth tumor region, and *B* represents the model’s predicted tumor region. $|A \cap B|$ denotes the number of overlapping voxels between the ground truth and the prediction, while |*A*| and |*B*| represent the number of voxels in the ground truth and predicted regions, respectively. The Intersection over Union (IoU), also known as the Jaccard index, is another common metric for evaluating segmentation quality. Similar to the Dice score, it measures the overlap, but it is calculated as the ratio of the intersection to the union. Its value also ranges from [0, 1], with values closer to 1 indicating better performance. The formula is:

18 $$\begin{aligned} \text {IoU} = \frac{|A \cap B|}{|A \cup B|} \end{aligned}$$

where $|A \cup B|$ represents the number of voxels in the union of the ground truth and predicted regions, i.e., $|A| + |B| - |A \cap B|$.

The 95% Hausdorff Distance (HD95) is a variant of the Hausdorff Distance (HD) specifically designed to reduce errors caused by outliers in distance calculation. The formula for HD is:

19 $$\begin{aligned} HD(A, B) = \max (h(A, B), h(B, A)) \end{aligned}$$

where *h*(*A*, *B*) and *h*(*B*, *A*) represent the directed distances from point set A to point set B and vice versa. HD95 discards the 5% of points with the largest distances when calculating the distances between point sets; otherwise, its calculation is identical to that of HD. In medical image segmentation, HD95 is a more stable and robust evaluation metric than HD. A lower HD95 value indicates better model performance. Simultaneously, we employed Accuracy, Precision, Recall, and the Area Under the Receiver Operating Characteristic Curve (AUC) as the primary metrics for evaluating the model’s classification performance. These metrics assess the model’s classification effectiveness from different perspectives. Accuracy reflects the overall correctness of predictions, Precision measures the reliability of positive class predictions, Recall evaluates the model’s ability to identify positive classes, and AUC quantifies the model’s overall classification performance across various thresholds.

**Table 2:** Experimental environment and key dependencies

Component / Dependency	Version	Purpose
Python	3.8.18	Programming language for the experimental environment
PyTorch	2.6.0	Deep learning framework for model training
NumPy	1.26.4	Library for numerical computation and tensor manipulation
OpenCV	4.11.0.86	Computer vision library for image processing
CUDA	12.8	Platform for GPU-accelerated computing
GPU	NVIDIA RTX A6000	Hardware to accelerate deep learning training and inference

### Experimental environment and procedure

Our experimental environment, along with the major dependencies and their corresponding version numbers, is detailed in Table 2. During model training, the initial learning rate was set to 0.0001 for all models, and each model was trained for 250 epochs to ensure full optimization of the model parameters and achieve optimal final performance. The training batch size was set to 16. All models and modules were trained using the Adam optimizer to enhance convergence speed and optimization effectiveness. Furthermore, to optimize the training process, we incorporated a cosine annealing learning rate scheduler. This strategy maintains a relatively large learning rate in the initial stages of training to promote rapid convergence and then gradually decreases the learning rate as training progresses. This improves the stability of the training process, helps to avoid overfitting, and ensures that a more optimal model performance is ultimately achieved.

### Comparative experiments

To validate the segmentation performance of our proposed method on the ABVS dataset, we selected six representative network models for comparison. Among these, U-Net3D represent classic 3D segmentation networks whose mature encoder-decoder architectures have been widely validated in the field of medical image segmentation. SegResNet incorporates the concept of residual learning, effectively mitigating the vanishing gradient problem in deep networks through residual blocks, thereby optimizing the feature extraction process. nnU-Net, as a self-adapting medical segmentation framework, supports 2D, 3D U-Net, and cascaded architectures; it automatically adapts to the characteristics of different datasets by dynamically adjusting network topology, patch sampling strategies, and data augmentation schemes to achieve more stable and precise segmentation. Meanwhile, UNETR and SegFormer3D represent the recent trend of Transformer based or hybrid architecture models. These models leverage global self-attention mechanisms or combine the advantages of local convolutional features to excel at capturing both global context and fine-grained local details. Furthermore, we include MedSAM2 as a benchmark to evaluate our specialized architecture against a state-of-the-art foundation model. To ensure a fair comparison, we conducted full-parameter fine-tuning on MedSAM2 using a slice-by-slice training strategy. Specifically, 3D volumes were decomposed into 2D axial slices, and during the training phase, positive point prompts were randomly sampled from the ground truth masks to supervise the prompt-to-mask mapping. In the inference stage, a single positive point located at the centroid of the target was provided for each slice to ensure deterministic and reproducible results. In summary, these six network models cover a range of design philosophies, from traditional CNNs to modern Transformer, hybrid, and foundation architectures, providing a comprehensive and effective benchmark to evaluate the strengths and weaknesses of our proposed method.The experimental results are shown in Table 3.

**Table 3:** Comparison of segmentation performance with state-of-the-art methods. Best results are in **bold**

Network Model	Dice (%)$\uparrow$	IoU (%)$\uparrow$	HD95 (mm)$\downarrow$
U-Net3D [17]	78.79 ± 0.26	75.75 ± 0.32	7.91 ± 2.12
SegResNet [18]	76.53 ± 0.25	71.88 ± 0.27	8.72 ± 1.93
nnU-Net [19]	80.62 ± 0.18	77.67 ± 0.23	7.87 ± 1.65
UNETR [20]	79.11 ± 0.21	76.10 ± 0.26	8.32 ± 1.68
Segformer3D [21]	81.66 ± 0.17	78.03 ± 0.21	7.74 ± 1.73
MedSam2 [22]	80.84 ± 0.19	77.89 ± 0.24	7.80 ± 1.69
Ours	**83.93** **± 0.22**$^{*}$	**79.33** **± 0.24**$^{*}$	**7.28** **± 0.85**$^{*}$

$^{*}$indicates$p < 0.05$compared with the second-best model (Segformer3D) via Wilcoxon signed-rank test

As can be seen from the experimental results in the table, our proposed method demonstrated a significant advantage across all metrics in the segmentation comparison on the ABUS-TDSC dataset, fully proving its outstanding performance in medical image segmentation tasks. According to the quantitative results, our method achieved a Dice score of 83.93% and an IoU of 79.33%, both of which surpassed all competing models. Compared to the second-best model, Segformer3D, our method improved the Dice score by approximately 2.27 percentage points and the IoU by 1.3 percentage points, a marked improvement. Furthermore, the gap between our method and nnU-Net widened to 3.31 percentage points in the Dice score, confirming the robustness and reliability of our model’s segmentation accuracy.

In addition to its excellent segmentation performance, the superiority of our method is also reflected in its boundary delineation capability. The HD95 metric was 7.28mm, a reduction of approximately 1.55% compared to Segformer3D’s 7.74mm and a substantial 8.41% reduction compared to UNETR’s 8.32mm. We speculate that the Boundary-Aware Feature Fusion Module enhanced the model’s understanding of lesion boundaries, making our method more stable in capturing the details of complex anatomical structures and handling boundaries. Thanks to this module, the model can not only segment the target area more accurately but also perceive the contour and morphology of the tumor more precisely, which implicitly enhances its clinical usability.

Overall, our proposed method excels in overall segmentation accuracy, regional consistency, and boundary detail processing, demonstrating good generalization ability and application potential. To more intuitively display the segmentation results of each model, we have selected and visualized the results from four images (Fig.8).

To comprehensively evaluate the effectiveness of our proposed method, we further selected six representative 3D classification models for comparison, building upon the segmentation experiments. Among them, ResNet3D, as a classic 3D CNN structure widely used in 3D medical image analysis, serves as a representative baseline model with strong stability and reproducibility. EfficientNet3D, a lightweight 3D CNN, maintains excellent classification performance at a lower computational cost, making it particularly suitable for resource-constrained environments and small-scale datasets. Within the Transformer family, MedFormer is a model specifically designed for medical imaging tasks, possessing powerful global information modeling capabilities to effectively capture long-range dependencies, showing excellent performance in handling complex medical image data. SwinTransformer3D is also based on the Transformer architecture and introduces a sliding window attention mechanism, enabling it to model multi-scale features more efficiently, thus performing well in many medical image classification tasks. Additionally, we selected the hybrid architecture CoAtNet3D and the optimized CNN structure ConvNeXt3D for comparison. CoAtNet3D combines the advantages of both CNNs and Transformers, leveraging the efficiency of CNNs in feature extraction and the global modeling capabilities of Transformers to achieve superior performance in classification tasks. ConvNeXt3D is an efficient network that optimizes traditional CNNs, striking a good balance between computational cost and performance, and narrowing the gap between CNNs and Transformers to some extent.

**Table 4:** Comparison of classification performance with state-of-the-art methods. Best results are in **bold**

Network Model	Accuracy (%)	AUC (%)	Precision (%)	Recall (%)	Specificity (%)
ResNet3D [23]	81.25 ± 0.15	82.98 ± 0.27	78.25 ± 0.18	77.67 ± 0.12	80.05 ± 0.25
EfficientNet3D [24]	82.47 ± 0.18	84.02 ± 0.20	79.31 ± 0.29	**81.20** **± 0.17**	77.51 ± 0.19
Swin Transformer3 [25]	73.53 ± 0.36	76.44 ± 0.12	72.06 ± 0.22	73.92 ± 0.14	75.41 ± 0.28
MedFormer [26]	74.19 ± 0.21	75.22 ± 0.28	74.02 ± 0.27	71.68 ± 0.18	77.97 ± 0.15
CoAtNet3D [27]	78.27 ± 0.21	79.35 ± 0.22	77.11 ± 0.18	76.50 ± 0.24	79.32 ± 0.21
ConvNeXt3D [28]	80.62 ± 0.29	82.49 ± 0.23	**80.04** **± 0.17**	78.38 ± 0.23	81.15 ± 0.22
Ours	**82.93** **± 0.16**$^{*}$	**84.55** **± 0.19**$^{*}$	79.84 ± 0.22	79.77 ± 0.13	**82.01** **± 0.22**$^{*}$

$^{*}$indicates statistical significance ($p < 0.05$) compared with the second-best model

In summary, these six models each have distinct characteristics, including classic CNN structures, lightweight and efficient CNNs, highly optimized CNN variants, as well as Transformer and CNN-Transformer hybrid architectures specifically optimized for medical imaging. This provides a comprehensive and thorough comparative evaluation for our method. The experimental results are shown in Table 4. The results of the classification comparison on the ABUS-TDSC dataset indicate that our proposed method shows significant advantages across multiple evaluation metrics, with particularly outstanding performance in accuracy and AUC.

In terms of overall performance, our method achieved an accuracy of 82.93%, surpassing all competing models. It showed an improvement of 0.46% over the second-place EfficientNet3D, and 1.68% and 2.31% over ResNet3D and ConvNeXt3D, respectively, demonstrating a stronger overall classification capability. This improvement suggests that our method maintains more stable recognition performance across different types of breast lesions, reducing misjudgments caused by data distribution biases or image complexity. Furthermore, on the AUC metric, which measures the model’s ability to distinguish between benign and malignant samples, our method achieved an optimal score of 84.55%, far exceeding all comparison models. Compared to the second-best AUC score from ConvNeXt3D, our method showed an improvement of 2.06%, and a 1.57% improvement over ResNet3D. The improvement in this metric reflects the stability of our model across different decision thresholds and further proves its superior global discriminative power in distinguishing between benign and malignant samples, which is of great significance for clinical applications. An increased AUC indicates that our method can effectively reduce the false detection rate while maintaining high sensitivity, making it more suitable for clinical scenarios. In contrast, Swin Transformer3D and MedFormer had AUC scores of 76.44% and 75.22%, respectively, both significantly lower than the other models, suggesting limitations in their feature extraction and classification capabilities on ABVS images. We speculate this may be due to the small dataset size.

Other metrics such as precision, recall, and specificity also play important roles in classification performance. Overall, our method achieved a good balance among these three metrics. In contrast to some models that showed a bias towards one metric (e.g., Swin Transformer3D had declines in both precision and recall, and MedFormer had a lower recall, increasing the risk of missed diagnoses), our method demonstrates strong stability while ensuring overall classification accuracy. It is particularly noteworthy that our method’s specificity reached 82.01%, an improvement of 1.96% and 0.86% over ResNet3D and ConvNeXt3D, respectively. This indicates its advantage in reducing false positives, which helps to avoid additional examinations due to misdiagnosis and reduces the psychological burden on patients. In summary, our proposed method demonstrates excellent overall performance in the 3D medical image classification task for breast cancer, with its accuracy and AUC significantly outperforming other comparative models. This fully validates the effectiveness of our proposed method in classifying benign and malignant breast lesions.

### Ablation study

To investigate the effectiveness of the proposed multi-task architecture, we designed two sets of ablation experiments: a multi-task architecture ablation study and a module ablation study. These are described below.

#### Multi-task architecture ablation study

To comprehensively evaluate the effectiveness of the proposed multi-task architecture for ABVS images, we designed and conducted an ablation study to investigate the roles of the segmentation and classification branches in the overall model performance. The experiment was divided into three groups: a model with only the segmentation branch (S), a model with only the classification branch (C), and the complete multi-task segmentation and classification architecture (S+C / Ours). The complete architecture integrates both tasks to validate the enhancement in feature extraction and decision-making capabilities, as well as the correlation between the two tasks. To ensure the objectivity and comparability of the results, all experiments used the same data preprocessing pipeline and were evaluated under the same training and testing environments. The results are shown in Table 5. In this set of ablation experiments, by comparing the results of the segmentation-only branch (S), the classification-only branch (C), and the complete multi-task architecture (Ours), the complementary effects of multi-task learning and the significant improvement in overall performance are clearly visible. Specifically, the single-task segmentation model achieved a Dice score of 80.77%, an IoU of 78.46%, and an HD95 of 7.91. The single-task classification model achieved an accuracy of 78.35%, an AUC of 80.21%, a precision of 79.92%, a recall of 77.12%, and a specificity of 79.44%. In contrast, the multi-task model, which integrates both segmentation and classification, showed marked improvements on both tasks. For the segmentation task, the Dice score increased from 80.77% to 83.93%, an increase of approximately 3.9%, which is a significant gain. The IoU also rose from 78.46% to 79.33%; although the increase is smaller, it still reflects an improved boundary prediction capability. Meanwhile, the HD95 decreased from 7.91 to 7.28, a reduction of about 3.7%, indicating more refined segmentation results. We speculate this is because the model’s Boundary-Aware Feature Fusion Module effectively extracted boundary information and integrated it into the segmentation branch. For the classification task, the multi-task model’s accuracy jumped from 78.35% to 82.93%, an increase of about 5.8%, and the AUC rose from 80.21% to 84.55%, an increase of about 5.4%. Although there was a slight decrease in precision compared to the single-task classification model, the multi-task model improved recall and specificity by 2.65 and 2.57 percentage points, respectively, indicating that the overall discriminative ability of the model was enhanced. This series of improvements fully demonstrates that the proposed multi-task architecture can effectively integrate the classification and segmentation tasks, thereby more efficiently sharing and utilizing feature information to promote the performance of each individual task and achieve a positive interaction between them.

**Table 5:** Ablation study of the multi-task architecture. ‘S‘ denotes the segmentation-only model, ‘C‘ denotes the classification-only model, and ‘Ours‘ is the proposed multi-task model. Best results are in **bold**

Experiment	Segmentation Metrics			Classification Metrics				
	Dice (%)$\uparrow$	IoU (%)$\uparrow$	HD95 (mm)$\downarrow$	Accuracy (%)$\uparrow$	AUC (%)$\uparrow$	Precision (%)$\uparrow$	Recall (%)$\uparrow$	Specificity (%)$\uparrow$
S (Seg-only)	80.77± 0.25	78.46± 0.27	7.91± 1.62	−	−	−	−	−
C (Cls-only)	−	−	−	78.35± 0.25	80.21± 0.21	**79.92** **± 0.15**	77.12± 0.23	79.44± 0.19
Ours (S+C)	**83.93** **± 0.22**$^{*}$	**79.33** **± 0.24** $^{*}$	**7.28** **± 0.85** $^{*}$	**82.93** **± 0.16** $^{*}$	**84.55** **± 0.19** $^{*}$	79.84 ± 0.22	**79.77** **± 0.13** $^{*}$	**82.01** **± 0.22** $^{*}$

$^{*}$indicates$p < 0.05$compared with the respective single-task baselines (S or C)

#### Module ablation study

To validate the effectiveness of the proposed MVHA and BAFFM, we conducted a comprehensive module ablation study. As shown in Table 6, the experiment was categorized into three levels: the baseline model (Baseline); a detailed decomposition of the MVHA module into Channel (C), Spatial (S), and Voxel (V) components; and the integration of the full MVHA and BAFFM (Ours).Effectiveness of MVHA and its Internal Components: From the perspective of segmentation, the baseline model achieved a Dice score of 79.12%. To further investigate the MVHA module, we analyzed its internal attention mechanisms. The results indicate that the individual incorporation of Channel, Spatial, and Voxel attention branches leads to incremental improvements, with Dice scores rising to 80.25%, 80.58%, and 80.91%, respectively. Notably, the Full MVHA configuration achieves a Dice of 81.70%, demonstrating that capturing multi-view dependencies (channel-wise features, spatial localization, and 3D voxel correlations) provides a synergistic effect. In terms of classification, the MVHA module significantly boosts the AUC from 80.02% (Baseline) to 83.47%, proving its superior ability to extract discriminative features for benign-malignant diagnosis.Effectiveness of BAFFM: The addition of the BAFFM to the baseline yielded an even more pronounced improvement in segmentation, with the Dice score rising impressively to 83.04%. The HD95 also decreased from 8.02 mm to 7.71 mm, confirming that explicitly supervising boundary-aware features significantly enhances the model’s perception of tumor lesion contours. For classification, the BAFFM increased the accuracy to 81.46%, indicating that accurate boundary information provides crucial morphological cues that assist the classifier in identifying lesion types.Synergy of All Modules (Ours): Finally, when both modules were combined (Ours), the framework achieved peak performance across almost all metrics. The Dice score reached 83.93% (a 6.1% relative improvement over baseline), the IoU rose to 79.33%, and the HD95 dropped significantly to 7.28 mm. Similarly, the classification accuracy and AUC reached their highest values at 82.93% and 84.55%, respectively. The statistical significance ($p < 0.05$) highlighted in Table 6 further proves that the Medical Voxel Hybrid Attention module and the Boundary-Aware Feature Fusion Module have a strong complementary effect. While MVHA refines feature representation through multi-view attention, BAFFM ensures structural integrity through boundary awareness, jointly contributing to the model’s overall excellence in both tasks.

**Table 6:** Detailed ablation study of the proposed modules. MVHA is further decomposed into Channel (C), Spatial (S), and Voxel (V) attention components to verify their individual contributions. Best results are in **bold**

Experiment	Segmentation Metrics			Classification Metrics				
	Dice (%)	IoU (%)	HD95 (mm)	Accuracy (%)	AUC (%)	Precision (%)	Recall (%)	Specificity (%)
Baseline	79.12± 0.27	77.46± 0.26	8.02± 1.71	78.74± 0.29	80.02± 0.24	76.43± 0.19	77.25± 0.25	75.31± 0.22
Baseline + MVHA (C)	80.25± 0.21	77.85± 0.22	8.01± 1.68	79.41± 0.22	81.33± 0.21	77.58± 0.20	78.02± 0.19	77.42± 0.24
Baseline + MVHA (S)	80.58± 0.24	77.98± 0.19	7.95± 1.65	79.62± 0.18	81.85± 0.23	78.12± 0.23	78.44± 0.22	78.15± 0.21
Baseline + MVHA (V)	80.91± 0.20	78.11± 0.25	7.89± 1.60	79.88± 0.25	82.14± 0.19	78.45± 0.18	78.61± 0.24	78.86± 0.23
Baseline + MVHA (Full)	81.70± 0.23	78.23± 0.20	7.82± 1.63	80.33± 0.19	83.47± 0.25	79.06± 0.25	78.99± 0.21	79.82± 0.20
Baseline + BAFFM	83.04± 0.22	78.95± 0.31	7.71± 1.44	81.46± 0.23	83.25± 0.19	78.65± 0.21	78.46± 0.27	81.78± 0.23
Ours (All modules)	**83.93** ± **0.22**$^{*}$	**79.33**± **0.24**$^{*}$	**7.28**± **0.85**$^{*}$	**82.93**± **0.16**$^{*}$	**84.55**± **0.19**$^{*}$	**79.84** ± **0.22**$^{*}$	**79.77**± **0.13**$^{*}$	**82.01**± **0.22**$^{*}$

$^{*}$indicates$p < 0.05$compared with the best-performing ablation variant

### Cross-dataset generalization analysis

To assess the robustness and clinical transferability of our framework, we conducted cross-dataset experiments by training on one domain and performing "zero-shot" evaluation on the other (i.e., public-to-private and private-to-public assessments). This setup directly evaluates the model’s ability to mitigate domain shift caused by variations in imaging protocols and scanner manufacturers. As summarized in Table 7, although a performance decay is observed when moving from internal to external evaluation—such as a Dice score decrease from 85.23% to 79.54% when generalizing from TDSC-ABUS to the private dataset—the model maintains a high Dice score (>77%) and AUC (>80%) in both directions. This stability confirms that our boundary-aware multi-task learning framework successfully captures domain-invariant morphological features rather than overfitting to scanner-specific artifacts. Notably, classification metrics remain resilient even during external testing, suggesting that the shared feature representations are highly robust. In summary, the cross-dataset validation demonstrates that our proposed framework possesses strong generalization capabilities, making it a promising candidate for deployment in diverse clinical environments.

**Table 7:** Cross-dataset generalization analysis. Internal results refer to the test split of the training domain, while external results indicate zero-shot generalization to the other dataset. Values are reported as Mean ± SD (%)

Training Domain	Testing Domain	Dice$\uparrow$	IoU$\uparrow$	Accuracy$\uparrow$	AUC$\uparrow$
TDSC-ABUS (Public)	Internal (Public Test)	85.23 ± 0.31	81.82 ± 0.44	83.51 ± 0.22	85.14 ± 0.35
	External (Private Set)	79.54 ± 0.52	76.21 ± 0.61	80.12 ± 0.48	82.37 ± 0.52
Private Dataset	Internal (Private Test)	83.12 ± 0.45	78.53 ± 0.52	82.25 ± 0.31	84.22 ± 0.47
	External (Public Set)	77.87 ± 0.74	74.92 ± 0.83	78.41 ± 0.62	80.51 ± 0.76

## Discussion

In this work, we have proposed a deep learning architecture based on multi-task learning for the intelligent analysis of ABVS images. To address the specific characteristics of breast tumor segmentation and classification, we introduced a series of effective innovations to enhance the model’s overall performance. First, we constructed a unified multi-task learning framework that allows for the synergistic optimization of tumor region segmentation and benign-malignant classification within a single network. This approach fully leverages the complementary information between the two tasks, thereby improving both the model’s understanding of the lesion area and the accuracy of tumor classification. During the decoding process, we introduced an auxiliary boundary segmentation task and designed a Boundary-Aware Feature Fusion Module (BAFFM) to acquire and fully utilize boundary semantic information. This enables the integration of the tumor’s contour information into the final decision-making for both segmentation and classification, which further strengthens the intrinsic connection between the two tasks, allowing the model to delineate lesion boundaries more precisely while extracting more discriminative features. Additionally, we proposed a Medical Voxel Hybrid Attention (MVHA) mechanism. This attention mechanism effectively fuses spatial and channel information and adaptively adjusts feature representations, enabling the model to identify lesions more effectively and improve classification stability against complex breast tissue backgrounds. To comprehensively validate the effectiveness of our proposed method, we conducted extensive comparative and ablation experiments on the TDSC-ABUS dataset. In the comparative experiments, our proposed model significantly outperformed existing methods across several core evaluation metrics, including AUC, accuracy, and Dice score, fully demonstrating its superior performance in medical image analysis tasks. In the ablation studies, we investigated the individual contributions of the multi-task architecture, the MVHA module, and the BAFFM module to the overall model performance. The experimental results indicated that the multi-task learning architecture effectively integrates key information required for both segmentation and classification, promoting information sharing between them and thereby enhancing the model’s comprehensive performance. Simultaneously, both the MVHA and BAFFM modules played crucial roles; they respectively helped to improve the model’s perception of local details and lesion boundaries, further enhancing the precision of tumor region segmentation and the reliability of classification. In summary, the multi-task learning framework and its key modules that we have proposed provide an effective solution with significant clinical application value for the intelligent analysis of ABVS images.
